# Supplementary material for: White Matter Correlates of Early-Onset Bipolar Illness and Predictors of One-Year Recurrence of Depression in Adults with Bipolar Disorder
Source: J Clin Med. 2022 Jun 15;11(12):3432. doi: 10.3390/jcm11123432 (PMC9225103; doi:10.3390/jcm11123432)
Supplement: Supplementary file 1 [file jcm-11-03432-s001.zip › jcm-1746308-supplementary.pdf]

SUPPLEMENTAL MATERIAL.

METHODS.

Participants.

Exclusion criteria were: history of head injury, neurological disorders, neurodevelopmental disorders, serious medical illness, contraindication to participating in MRI (e.g., pregnancy, claustrophobia, or presence of metal in his/her body), history of any substance abuse in the last three months, and intoxication or use of illicit substances (except cannabis) in urine tests on the day of the scan.

Demographic and clinical characteristics of included and excluded BD participants are detailed in Table S1. There was no demographic or clinical difference between included and excluded BD participants.

Clinical assessments.

DSM-IV criteria used and corresponding PSR scores are reported in Table S2. Demographic and clinical differences between BD participants with and without post-scan longitudinal follow-up are detailed in Table S3. Participants lost during follow-up showed lower MASQ90 Anxious arousal scores and no use of mood stabilizers at scan.

Demographic and clinical differences between BD participants with and without post-scan recurrence of depression are detailed in Table S4. All participants were in full or partial remission (31 showed no or minimal symptoms, PSR 1-2; 21 showed only few weeks of subsyndromal symptoms, PSR 3-4). Participants with recurrence of depression one year after scan (Depressed BD) showed higher proportion of Caucasians, higher BIS total score, higher pre-scan percentage of syndromic and sub-syndromic depression.

Additional clinical and demographic measures.

Demographic variables included age, sex, employment status, educational level, race, and handedness. Specifically, sex consisted of two levels (male and female), employment status consisted of

three levels (unemployed, employed, and full-time student), educational level consisted of two levels (less than college degree and college degree), race consisted of two levels (Caucasian and non-Caucasian), and handedness consisted of two levels (left and right). Comorbid psychiatric disorders were classified as dichotomic (YES/NO) measures and included the following disorders: anxiety, psychotic, personality, developmental, substance use, and ADHD. Pharmacological treatment at scan was classified as YES/NO measures and included the following classes: antidepressants, antipsychotics, and mood stabilizers.

Neuroimaging data.

Acquisition.

Images were acquired on a 3T Siemens Prisma at the Magnetic Resonance Research Center, University of Pittsburgh Medical Center Health System, USA. A standard body coil was used for RF transmission, while MRI signal was acquired with a Siemens 64 RF channels receiver. Anatomical images covering the entire brain were acquired using an axial 3D MPRAGE sequence (TE/TI/TR=3.29ms/900ms/2200ms; flip angle=9; 192 1mm-thick slices; matrix size=256x192; time: 7’02”). A single-shot spin-echo planar imaging (SE-EPI) sequence was acquired with 210 optimized non-colinear diffusion-weighting gradient directions (35 volumes with b=700 s/mm2, 70 volumes with b= 1000 s/mm2, and 105 volumes with b= 2500 s/mm2) and 13 reference volumes with b=0 s/mm2 (repetition time (TR)= 8400ms, echo time (TE)= 90ms, flip angle= 90, field-of-view (FOV)= 256x256, sixty-four 2mm thick slices, no gaps, matrix size= 128x128, acquisition time= 10’). In accordance with a forward-reverse protocol, the acquisition was collected twice with opposite phase encoding directions (P>>A and A>>P).

Preprocessing.

Diffusion-weighted images were corrected for eddy current, subject motion and EPI distortion using topup and eddy [1,2], within FMRIB's Software Library (FSL). Six movement parameters, including average volume-by-volume translation and rotation in the x, y and z plane, were computed as previously proposed [3] in each participant. Absolute values were first derived for each parameter. Then, averaged

translation and averaged rotation were extracted for each participant to examine if there was a main effect of movements on main findings. A multi-compartment diffusion model was fitted to the data and used to estimate the probability distributions of our a-priori white matter tracts was based on the Bayesian framework for global tractography proposed in TRACULA [4]. Reconstruction of 16 major white matter tracts was performed using TRActs Constrained by UnderLying Anatomy (TRACULA) [4], in the FreeSurfer [5] package. Anatomical priors for tractography were derived from structural images using FreeSurfer. Tractography was performed in native space using a triple tensor model [6] and included the following white matter tracts: forceps minor, forceps major, 12 long associative tracts (left and right cingulum bundle, superior longitudinal fasciculus temporal, superior longitudinal fasciculus parietal, inferior longitudinal fasciculus, corticospinal tract and uncinate fasciculus), and two projective fibers (left and right anterior thalamic radiation). FA was then extracted from the 16 reconstructed tracts of interest. For each tract an overall mean FA value and nodal FA values were extracted to depict the collinearity of the fibers across (mean) and along (tract-profile of consecutive nodes) the entire tract. It is noteworthy mentioning that the number of nodes in each tract-profile depends on the different length of each tract and on inter subject variability of brain anatomy. So, for group-level analyses using tract-profile measures, nodal measures representing each white matter pathway were interpolated to a fixed number (100 nodes) using Python. Overall mean FA and nodal FA for tract-profiles were extracted and imported into the Statistical Package for the Social Sciences (SPSS) for further statistical analyses. Using a similar approach, AD and RD values were also extracted to help interpret FA findings.

Statistical analyses

Level 1: primary hypothesis testing.

Thirty-three variables were included in the GLMNET model. Six demographic variables (age, sex, employment status, educational level, race, and handedness), seven before study entry variables (age of first BD episode, number of years with BD, socioeconomic status at study entry, and number of depressive,

manic, hypomanic, and mixed episodes before study entry), six psychiatric comorbidities (anxiety, psychotic, personality, developmental, substance use, and ADHD), three medications classes at scan (antidepressants, antipsychotics, and mood stabilizers), and 11 pre-scan percentages (syndromic depression, syndromic mania, syndromic hypomania, sub-syndromic depression, sub-syndromic hypomania, mixed symptoms, euthymia, antidepressants, antipsychotics, lithium, and non-lithium mood stabilizers). Age of first BD episode was included as a categorical variable to indicate the developmental stage in which participants had the first episode (childhood, early adolescence, or late adolescence). In addition, the number of years of BD was calculated based on the age of first BD episode and age at scan. Dummy variables were created to represent categorical variables with more than two levels.

RESULTS.

Level 1: primary hypothesis testing.

Focal abnormalities in tracts of interest.

Table S5 shows the contribution of each predictor identified in Level 1 analysis to explain the FA variability in each white matter tract that survived FDR correction. The FA variability in the FMIN was best explained by the number of depressive episodes during childhood/adolescence (43.1%) while the FA variability in both left and right CB was best explained by percentage of time experiencing syndromic depression (53.7% and 50.7% respectively).

Table S6 shows the effects of the clinical model identified in Level 1 analysis in AD and RD. There was no association between the clinical model and AD or RD in these node clusters.

Relationship between focal abnormalities and symptoms and medications at scan

Table S7 shows relationships between mean FA of node clusters identified in Level 1 analysis and 13 symptom dimensions collected at scan. Lower FA in the FMIN middle left node cluster was correlated

with higher ALS, MASQ90 General distress depressive, MASQ90 General distress mixed, and STAIY Trait scores. Lower FA in the other three node clusters (FMIN middle right cluster, left CB anterior cluster, and right CB anterior cluster) was correlated with higher MASQ90 General distress depressive and STAIY Trait scores. There was no correlation between nodes FA and other symptoms at scan.

Table S8 shows the effects of medications classes at scan in the mean FA of node clusters identified in Level 1 analysis. There was no effect of medications at scan on the mean FA of these four node clusters.

Level 2: Secondary hypothesis testing.

The BIS total score was identified as a predictor of future depressive episodes in Level 2 analysis. Among the BIS second order factors (attentional, motor, and nonplanning impulsiveness), only higher attentional impulsiveness (B=1.02, Wald=7.85, P=0.005, FDR P=0.015, AOR=2.8, 95%CI= [1.37-5.82]) was associated with increased risk of recurrence of depression. Motor (B=0.47, Wald=2.36, P=0.124, FDR P=0.158, AOR=1.6, 95%CI= [0.88-2.93) and nonplanning (B=0.43, Wald=21.99, P=0.158, FDR P=0.158, AOR=1.5, 95%CI= [0.85-2.81]) impulsiveness were not associated.

Exploratory analyses.

Table S9 shows the between group differences in tracts or other node clusters included but not selected in Level 2 analysis. Depressed BD showed lower FA in the left CB anterior cluster in comparison to Non-depressed BD (F=5.0, P=0.030, FDR P=0.090). However, this finding did not survive FDR correction. There were no other between group differences.

Table S10 shows the effects of dMRI movements (translation and rotation) in node clusters identified in Level 1 analysis. There was no correlation between translation or rotation and FA in tracts or node clusters included in Level 1 and 2 analysis.

Table S11 shows the effect of predictors identified in Level 1 analysis in other white matter tracts (forceps major; left/right anterior thalamic radiation, inferior longitudinal fasciculus, superior longitudinal fasciculus - temporal, superior longitudinal fasciculus – parietal, and corticospinal tract). The model

identified in Level 1 analysis was associated with lower FA in the forceps major and anterior thalamic radiation. However, these findings did not survive FDR correction

Table S12 shows the correlations between percentage of time taking medications pre-scan and mean FA of node clusters identified in Level 1 analysis. There was no significant correlation between pre-scan time taking medications and node clusters FA.

SUPPLEMENTAL TABLES.

Table S1. Clinical and demographic characteristics of BD participants included/excluded in Level 1 analysis.

Characteristics	Total (N=78)	Included BD (N= 70)	Excluded BD (N=8)	t(76) or χ2	P value[a]
Age (years), mean[SD]	26.5[3.9]	26.3 [3.9]	28.1[2.8]	-1.2	0.219
Sex, No. (%)					
Men	40 (51.3%)	35 (50.0%)	5 (62.5%)	0.5	0.503
Women	38 (48.7%)	35 (50.0%)	3 (37.5%)		
Educational Level,[b] No. (%)					
Higher	20 (25.6%)	14 (20.0%)	6 (75%)	0.1	0.740
Lower	58 (74.4%)	56 (80%)	2 (25%)		
Handedness, No. (%)					
Left	12 (15.4%)	11 (15.7%)	1 (12.5%)	0.1	0.811
Right	66 (84.6%)	59 (84.3%)	7 (87.5%)		
Race, No. (%)					
Caucasian	57 (73.1%)	52 (74.3%)	5 (62.5%)	0.5	0.476
Non-Caucasian	21 (26.9%)	18 (25.7%)	3 (37.5%)		
Employment Status, No. (%)					
Employed	57 (73.1%)	50 (71.4%)	7 (87.5%)		
Unemployed	15 (19.2%)	14 (20.0%)	1 (12.5%)	1.1	0.563
Full-time student	6 (7.7%)	6 (8.6%)	0 (0.0%)		
Clinical characteristics at-scan, mean[SD]					
HDRS	10.2[6.9]	9.8[6.5]	14.3[9.0]	-1.8	*0.080*
YMRS	3.9[3.3]	3.9[3.1]	4.5[4.7]	-0.5	0.609
BIS	65.3[12.0]	64.9[12.2]	68.1[10.7]	-0.7	0.480
ALS	53.1[38.4]	52.9[37.5]	55.0[48.5]	-0.1	0.882
SSS	17.2[6.2]	17.2[6.2]	17.1[7.1]	<0.1	0.991
STAIY State total	38.3[10.8]	38.1[10.3]	40.1[15.9]	-0.5	0.633
STAIY Trait total	40.8[11.1]	41.1[10.8]	38.3[14.8]	0.7	0.493
MASQ90 Anhedonic depression	60.1[14.0]	60.5[14.5]	56.0[8.3]	0.9	0.392
MASQ90 Anxious arousal	23.4[10.3]	22.5[8.4]	31.1[19.7]	-1.2	0.258
MASQ90 Loss of interest	14.4[6.1]	14.4[6.3]	15.1[5.3]	-0.3	0.740
MASQ90 General distress - Depressive	21.1[9.9]	20.9[9.8]	22.3[11.7]	-0.3	0.724
MASQ90 General distress - Anxious	18.1[7.2]	18.0[6.8]	19.3[11.8]	0.4	0.665

MASQ90 General distress - Mixed	32.4[12.0]	32.0[11.4]	35.8[17.3]	-0.8	0.411

Abbreviations: HC, Healthy controls; BD, Bipolar Disorder; HDRS, Hamilton Rating Scale for Depression; YMRS, Young Mania Rating Scale; BIS, Barratt Impulsiveness Scale; ALS, Affective Lability Scale; SSS, Sensation-Seeking Scale; STAIY, State-Trait Anxiety Inventory; MASQ90, Mood and Anxiety Symptom Questionnaire.

[a] P values ⩽ .05 are reported in bold characters.

[b] Educational level was defined as higher (college degree and above) and lower (high School or some college).

Table S2. Psychiatric Status Ratings (PSR) descriptions.

Code	Term	Description
6	Definitive Criteria (Severe)	Meets DSM-IV criteria for definite episode and has either prominent psychotic symptoms or extreme impairment in functioning.
5	Definitive Criteria (Severe)	Meets DSM-IV criteria for definite, current episode, but has no prominent psychotic symptoms or extreme impairment in functioning.
4	Marked	Does not meet definite DSM-IV criteria but has major symptoms or impairment from the disorder.
3	Partial Remission	Considerably less psychopathology than full criteria with no more than moderate impairment in functioning, but still has obvious evidence of the disorder. This category may represent a worsening or an improvement in the participant's prior status (e.g., a depressive episode with only 2 or 3 symptoms in a moderate degree, or 1 or 2 symptoms in a severe degree).
2	Residual	Either participant claims to not be completely back to “usual self”, or rater notes the presence of one or more symptoms of the disorder in no more than a mild degree (e.g., depressive episode with mild insomnia as only residual symptom).
1	Baseline	Participant returns to “usual self” without any residual symptoms of the disorder but may or may not have significant symptoms from some other condition or disorder (if so, this should be coded under that condition or disorder).

Abbreviations: DSM-IV, Diagnostic and Statistical Manual of Mental Disorders IV.

Table S3. Clinical and demographic characteristics of BD participants included/excluded in Level 2 analysis.

Characteristics	Total (N=70)	BD participants with post-scan follow-up (N= 52)	BD participants without post-scan follow-up (N=18)	t(68) or $\chi 2$	P value[a]
Age (years), mean[SD]	26.3 [3.9]	25.9[3.8]	27.5[4.1]	-1.5	0.149
Sex, No. (%)					
Men	35 (50.0%)	25 (48.1%)	10 (55.6%)	0.3	0.584
Women	35 (50.0%)	27 (51.9%)	8 (44.4%)		
Educational Level,[b] No. (%)					
Higher	14 (20.0%)	11 (21.2%)	3 (16.7%)	0.2	0.682
Lower	56 (80%)	41 (78.8%)	15 (83.3%)		
Handedness, No. (%)					
Left	11 (15.7%)	9 (17.3%)	2 (11.1%)	0.4	0.534
Right	59 (84.3%)	43 (82.7%)	16 (88.9%)		
Race, No. (%)					
Caucasian	52 (74.3%)	38 (73.1%)	14 (77.8%)	0.2	0.694
Non-Caucasian	18 (25.7%)	14 (26.9%)	4 (22.2%)		
Employment Status, No. (%)					
Employed	50 (71.4%)	37 (71.2%)	13 (72.2%)	2.8	0.245
Unemployed	14 (20.0%)	9 (17.3%)	5 (27.8%)		
Full-time student	6 (8.6%)	6 (11.5%)	0 (0.0%)		
Clinical characteristics at-scan, mean[SD]					
HDRS	9.8[6.5]	10.0[6.4]	9.1[6.9]	0.5	0.620
YMRS	3.9[3.1]	3.7[2.5]	4.3[4.5]	-0.5	0.522
BIS	64.9[12.2]	65.5[11.1]	63.3[15.2]	0.6	0.523
ALS	52.9[37.5]	56.5[37.6]	42.3[36.0]	1.4	0.169
SSS	17.2[6.2]	17.3[6.4]	16.7[5.6]	0.4	0.701
STAIY State total	38.1[10.3]	38.9[10.0]	35.6[11.0]	1.2	0.233
STAIY Trait total	41.1[10.8]	42.4[10.6]	37.6[10.5]	1.7	0.102
MASQ90 Anhedonic depression	60.5[14.5]	61.3[15.1]	58.3[12.8]	0.7	0.463
MASQ90 Anxious arousal	22.5[8.4]	23.4[9.4]	19.8[3.7]	2.3	**0.023**
MASQ90 Loss of interest	14.4[6.3]	14.9[6.7]	12.8[4.4]	1.5	0.135
MASQ90 General distress - Depressive	20.9[9.8]	21.8[10.7]	18.3[6.2]	1.3	0.195
MASQ90 General distress - Anxious	18.0[6.8]	18.7[7.2]	16.2[4.8]	1.3	0.190
MASQ90 General distress - Mixed	32.0[11.4]	33.1[11.7]	29.0[10.1]	1.3	0.193

Medications at-scan, Number of participants taking (%)					
Antidepressants	7 (10.0%)	6 (11.6%)	1 (5.6%)	0.5	0.466
Antipsychotics	9 (12.9%)	8 (15.4%)	1 (5.6%)	1.2	0.283
Mood stabilizers	10 (14.3%)	10 (19.2%)	0 (0.0%)	4.0	**0.044**
Psychiatric comorbidities reported at scan, No. (%)					
Anxiety disorders	36 (51.4%)	27 (51.9%)	9 (50.0%)	<0.1	0.888
Psychotic disorders	3 (4.2%)	3 (5.8%)	0 (0.0%)	1.1	0.298
Personality disorders	3 (4.2%)	2 (3.8%)	1 (5.6%)	0.1	0.758
Developmental disorders	9 (12.9%)	5 (9.6%)	4 (22.2%)	1.9	0.168
Substance use disorders	22 (31.4%)	15 (28.8%)	7 (38.9%)	0.6	0.429
ADHD	27 (28.6%)	22 (42.3%)	5 (27.7%)	1.2	0.275
Number of years with BD, mean [SD]	17.7[2.8]	17.4[2.6]	18.5[3.1]	-1.6	0.121
Socioeconomic status at study entry, mean [SD]	3.1[1.2]	3.1[1.2]	3.0[1.2]	0.3	0.771
Age of BD onset,[c] developmental stages, No (%)					
Childhood	35 (50.0%)	28 (53.8%)	7 (38.9%)		
Early adolescence	19 (27.1%)	13 (25.0%)	6 (33.3%)	1.2	0.550
Late adolescence	16 (22.9%)	11 (21.2%)	5 (27.8%)		
Number of mood episodes before study entry, mean [SD]					
Depression	0.8[1.5]	0.7[1.5]	1.3[1.5]	-1.4	0.163
Mania	1.2[3.7]	1.4[4.2]	0.6[1.9]	0.8	0.430
Hypomania	2.1[6.3]	2.4[7.1]	1.4[3.3]	0.6	0.571
Mixed episodes	0.3[0.5]	0.3[0.5]	0.3[0.5]	0.5	0.623
Pre-scan longitudinal measures, (mean % of follow-up time)					
Syndromic depression	5.8	6.1	5.0	0.5	0.620
Syndromic mania	0.4	0.4	0.5	-0.2	0.827
Syndromic hypomania	1.9	2.1	1.1	1.0	0.309
Sub-syndromic depression	12.0	12.4	11.0	0.5	0.594
Sub-syndromic hypomania	9.7	9.7	9.5	0.1	0.925
Mixed episodes	9.3	9.0	10.2	-0.4	0.701
Euthymia	60.9	60.2	62.6	-0.4	0.686
Antidepressants	15.4	17.4	9.8	1.7	0.101
Antipsychotics	31.3	32.1	28.9	0.4	0.701
Lithium	9.8	11.4	5.2	1.8	*0.077*
Non-lithium mood stabilizers	18.8	20.9	12.8	1.3	0.181

Abbreviations: BD, Bipolar Disorder; HDRS, Hamilton Rating Scale for Depression; YMRS, Young Mania Rating Scale; BIS, Barratt Impulsiveness Scale; ALS, Affective Lability Scale; SSS, Sensation-Seeking Scale; STAIY, State-Trait Anxiety Inventory; MASQ90, Mood and Anxiety Symptom Questionnaire.

[a] P values $\leqslant$ .05 are reported in bold characters.

[b] Educational level was defined as higher (college degree and above) and lower (high School or some college).

[c] The mean age of BD onset was 8.7[3.6]. There was no difference between these two groups.

Table S4. Clinical and demographic characteristics of BD participants included in Level 2 analysis (BD participants with and without post-scan depression).

Characteristics	**Total (N=52)**	**Non-depressed BD (N=33)**	**Depressed BD (N= 19)**	**t(50) or χ2**	**P value[a]**
Age (years), mean[SD]	25.9[3.8]	25.7[3.8]	26.3[4.0]	-0.5	0.626
Sex, No. (%)					
Men	25 (48.1%)	17 (51.5%)	8 (42.1%)	0.9	0.641
Women	27 (51.9%)	16 (48.5%)	11 (57.9%)		
Educational Level,[b] No. (%)					
Higher	11 (21.2%)	27 (81.8%)	14 (73.7%)	0.7	0.715
Lower	41 (78.8%)	6 (18.2%)	5 (26.3%)		
Handedness, No. (%)					
Left	9 (17.3%)	5 (15.2%)	4 (21.1%)	1.8	0.416
Right	43 (82.7%)	28 (84.8%)	15 (78.9%)		
Race, No. (%)					
Caucasian	38 (73.1%)	21 (63.6%)	17 (89.5%)	6.9	**0.031**
Non-Caucasian	14 (26.9%)	12 (36.4%)	2 (10.5%)		
Employment Status, No. (%)					
Employed	37 (71.2%)	21 (63.6%)	16 (84.2%)	3.8	0.428
Unemployed	9 (17.3%)	7 (21.2%)	2 (10.5%)		
Full-time student	6 (11.5%)	5 (15.2%)	1 (5.3%)		
Clinical characteristics at-scan, mean[SD]					
HDRS	10.0[6.4]	9.3[6.3]	11.3[6.5]	-1.1	0.284
YMRS	3.7[2.5]	3.3[2.3]	4.5[2.5]	-1.7	*0.097*
BIS	65.5[11.1]	62.3[10.0]	71.0[10.8]	-3.0	**0.005**
ALS	56.5[37.6]	52.5[36.0]	63.5[40.2]	-1.0	0.311
SSS	17.3[6.4]	16.4[6.3]	18.9[6.4]	-1.3	0.181
STAIY State total	38.9[10.0]	38.4[10.9]	39.9[8.5]	-0.5	0.609
STAIY Trait total	42.4[10.6]	40.5[10.8]	45.6[9.8]	-1.7	*0.099*
MASQ90 Anhedonic depression	61.3[15.1]	60.2[13.6]	63.2[17.6]	-0.7	0.487
MASQ90 Anxious arousal	23.4[9.4]	22.7[9.3]	24.7[9.8]	-0.7	0.462
MASQ90 Loss of interest	14.9[6.7]	13.5[5.5]	17.3[8.1]	-2.0	*0.054*
MASQ90 General distress - Depressive	21.8[10.7]	19.7[9.2]	25.5[12.3]	-1.9	*0.061*
MASQ90 General distress - Anxious	18.7[7.2]	17.8[7.5]	20.2[6.6]	-1.1	0.260
MASQ90 General distress - Mixed	33.1[11.7]	31.4[11.7]	36.0[11.4]	-1.4	0.175

Medications at-scan, Number of participants taking (%)					
Antidepressants	6 (11.6%)	3 (9.1%)	3 (15.8%)	1.5	0.474
Antipsychotics	8 (15.4%)	6 (18.2%)	2 (10.5%)	1.9	0.397
Mood stabilizers	10 (19.2%)	8 (24.2%)	2 (10.5%)	3.2	0.199
Psychiatric comorbidities reported at scan, No. (%)					
Anxiety disorders	27 (51.9%)	15 (45.5%)	12 (63.2%	2.5	0.291
Psychotic disorders	3 (5.8%)	1 (3.0%)	2 (10.5%)	1.9	0.401
Personality disorders	2 (3.8%)	1 (3.0%)	1 (5.3%)	0.5	0.794
Developmental disorders	5 (9.6%)	3 (9.1%)	2 (10.5%)	0.2	0.916
Substance use disorders	15 (28.8%)	9 (27.3%)	6 (31.6%)	2.4	0.307
ADHD	22 (42.3%)	11 (33.3%)	11 (57.9%)	3.0	0.225
Number of years with BD, mean [SD]	17.4[2.6]	17.1[2.3]	17.8[3.1]	-1.0	0.317
Socioeconomic status at study entry, mean [SD]	3.1[1.2]	2.9[1.2]	3.4[1.3]	-1.2	0.221
Age of BD onset,[c] developmental stages, No (%)					
Childhood	28 (53.8%)	17 (51.5%)	(51.5%)11		
Early adolescence	13 (25.0%)	9 (27.3%)	(27.3%)4	4.6	0.332
Late adolescence	11 (21.2%)	7 (21.2%)	(21.2%)4		
Number of mood episodes before study entry, mean[SD]					
Depression	0.7[1.5]	0.6[1.8]	0.8[1.0]	-0.3	0.732
Mania	1.4[4.2]	1.1[3.7]	2.1[4.9]	-0.8	0.416
Hypomania	2.4[7.1]	1.8[7.2]	3.5[7.2]	-0.8	0.409
Mixed episodes	0.3[0.5]	0.3[0.5]	0.4[0.6]	-0.2	0.817
Pre-scan longitudinal measures, (% of follow-up time)					
Syndromic depression	6.1[9.0]	3.0[5.4]	11.5[11.5]	-3.0	**0.006**
Syndromic mania	0.4[1.3]	0.4[1.3]	0.5[1.1]	-0.5	0.622
Syndromic hypomania	2.1[3.7]	2.4[4.3]	1.6[2.3]	0.8	0.447
Sub-syndromic depression	12.4[9.0]	10.2[8.7]	16.2[8.5]	-2.4	**0.019**
Sub-syndromic hypomania	9.7[9.6]	11.7[10.7]	6.4[6.2]	2.0	*0.055*
Mixed episodes	9.0[10.3]	9.5[10.8]	8.1[9.6]	0.5	0.649
Euthymia	60.2[21.3]	62.8[24.1]	55.7[14.5]	1.2	0.247
Antidepressants	17.4[27.3]	12.4[21.9]	26.0[33.7]	-1.6	0.128
Antipsychotics	32.1[29.8]	31.3[31.3]	33.6[27.7]	-0.3	0.791
Lithium	11.4[19.6]	11.0[18.9]	12.1[21.3]	-0.2	0.849
Non-lithium mood stabilizers	20.9[29.7]	19.3[30.7]	23.5[28.4]	-0.5	0.632

Abbreviations: BD, Bipolar Disorder; HDRS, Hamilton Rating Scale for Depression; YMRS, Young Mania Rating Scale; BIS, Barratt Impulsiveness Scale; ALS, Affective Lability Scale; SSS, Sensation-Seeking Scale; STAIY, State-Trait Anxiety Inventory; MASQ90, Mood and Anxiety Symptom Questionnaire.

[a] P values ⩽ .05 are reported in bold characters.

[b] Educational level was defined as higher (college degree and above) and lower (high School or some college).

[c] The mean age of BD onset was 8.6[3.8]. There was no difference between these two groups.

Table S5. Percentage of FA variability in each tract explained by the predictors identified in Level 1 analysis.

Pre-scan clinical predictor	White matter tracts		
	FMIN	Left CB	Right CB
Age at scan	8.7%	19.8%	38.1%
Caucasian	25.5%	21.1%	5.2%
Number of depressive episodes during childhood/adolescence	43.1%	5.5%	6.0%
Percentage of time experiencing syndromic depression	22.8%	53.7%	50.7%

Abbreviations: FMIN, Forceps Minor; CB, Cingulum bundle.

Table S6. Axial and Radial diffusivity results.

Table S6A. Axial diffusivity.

Node clusters	***F***	***P-value***[a]	***FDR P***[a,b]
FMIN middle left cluster	1.4	0.232	0.866
FMIN middle right cluster	0.5	0.752	0.866
Left CB - Anterior cluster	0.3	0.866	0.866
Right CB - Anterior cluster	0.6	0.678	0.866

Table S6B. Radial diffusivity.

Node clusters	***F***	***P-value***[a]	***FDR P***[a,b]
FMIN middle left cluster	<0.1	0.997	0.997
FMIN middle right cluster	0.5	0.455	0.997
Left CB - Anterior cluster	0.2	0.910	0.997
Right CB - Anterior cluster	0.9	0.855	0.997

Abbreviations: FMIN, Forceps Minor; CB, Cingulum bundle; FDR, False Discovery Rate.

[a] P values ≤ 0.05 are reported in bold characters.

[b] FDR corrected P values.

Table S7. Relationships between mean FA of node clusters and symptom dimensions at scan in Bipolar Disorder participants.

Table S7A. Forceps Minor node clusters.

Symptom dimensions	FMIN middle left cluster			FMIN middle right cluster		
	r	***P-value***[a]	***FDR P***[a,b]	***r***	***P-value***[a]	***FDR P***[a,b]
HDRS	-0.1	0.229	0.428	-0.2	0.180	0.428
YMRS	-0.2	*0.067*	0.268	-0.1	0.344	0.344
BIS	<0.1	0.815	0.815	-0.2	0.152	0.608
ALS	-0.3	**0.006**	**0.024**	-0.1	0.463	0.463
SSS	0.1	0.524	0.764	<0.1	0.832	0.832
STAIY State total	0.1	0.395	0.527	<0.1	0.887	0.887
STAIY Trait total	-0.4	**0.001**	**0.004**	-0.3	**0.005**	**0.010**
MASQ90 Anhedonic depression	-0.1	0.229	0.229	-0.2	**0.039**	0.120
MASQ90 Anxious arousal	-0.1	0.308	0.411	-0.1	0.275	0.411
MASQ90 Loss of interest	-0.2	*0.088*	0.176	-0.2	*0.078*	0.176
MASQ90 General distress - Depressive	-0.4	**0.001**	**0.004**	-0.3	**0.030**	**0.030**
MASQ90 General distress - Anxious	-0.3	**0.037**	0.148	-0.1	0.317	0.423
MASQ90 General distress - Mixed	-0.3	**0.005**	**0.020**	-0.2	0.146	0.195

Table S7B. Cingulum Bundle node clusters

Symptom dimensions	Left CB - Anterior cluster			Right CB - Anterior cluster		
	r	***P-value***[a]	***FDR P***[a,b]	***r***	***P-value***[a]	***FDR P***[a,b]
HDRS	-0.1	0.465	0.465	-0.1	0.321	0.428
YMRS	-0.1	0.335	0.344	-0.1	0.281	0.344
BIS	-0.1	0.377	0.754	-0.1	0.574	0.765
ALS	-0.3	**0.032**	*0.064*	-0.1	0.246	0.328
SSS	0.1	0.573	0.764	0.2	0.121	0.484
STAIY State total	-0.2	0.065	0.260	-0.1	0.351	0.527
STAIY Trait total	-0.3	**0.012**	**0.016**	-0.2	**0.041**	**0.041**
MASQ90 Anhedonic depression	-0.2	0.129	0.172	-0.2	*0.060*	0.120
MASQ90 Anxious arousal	-0.2	0.190	0.411	<0.1	0.955	0.955
MASQ90 Loss of interest	-0.1	0.392	0.523	-0.1	0.632	0.632
MASQ90 General distress - Depressive	-0.3	**0.009**	**0.018**	-0.3	**0.016**	**0.021**
MASQ90 General distress - Anxious	-0.2	0.113	0.226	-0.1	0.568	0.568
MASQ90 General distress - Mixed	-0.2	**0.046**	*0.092*	-0.1	0.234	0.234

Abbreviations: FMIN, Forceps Minor; CB, Cingulum bundle; FDR, False Discovery Rate; HDRS, Hamilton Rating Scale for Depression; YMRS, Young Mania Rating Scale; BIS, Barratt Impulsiveness Scale; ALS, Affective Lability Scale; SSS, Sensation-Seeking Scale; STAIY, State-Trait Anxiety Inventory; MASQ90, Mood and Anxiety Symptom Questionnaire.

[a] P values ≤ 0.05 are reported in bold characters.

[b] FDR corrected P values.

Table S8. Effect of medications at scan on mean FA of node clusters identified in Level 1 analysis.

Medications at scan	Antidepressants			Antipsychotics			Mood stabilizers		
	F	***P-value***[a]	***FDR P***[a,b]	***F***	***P-value***[a]	***FDR P***[a,b]	***F***	***P-value***[a]	***FDR P***[a,b]
FMIN middle left cluster	0.1	0.711	0.782	1.3	0.259	0.866	0.6	0.441	0.780
FMIN middle right cluster	0.7	0.398	0.782	0.2	0.651	0.866	0.6	0.448	0.780
Left CB - Anterior cluster	0.1	0.782	0.782	<0.1	0.866	0.866	0.3	0.585	0.780
Right CB - Anterior cluster	1.3	0.250	0.782	0.3	0.570	0.866	<0.1	0.840	0.840

Abbreviations: FMIN, Forceps Minor; CB, Cingulum bundle; FDR, False Discovery Rate.

[a] P values ≤ 0.05 are reported in bold characters.

[b] FDR corrected P values.

Table S9. Statistical results for exploratory between-group comparisons.

HC vs Depressed BD			
Node clusters identified in Level 1 analysis			
**Variable**	***F[1,54]***	***P*[a]**	***FDR P*[b]**
FMIN middle left cluster FA	1.8	0.188	0.338
Left CB – Anterior cluster FA	3.8	*0.058*	0.164
Right CB – Anterior cluster FA	0.6	0.450	0.556
Tracts or segments of tracts not associated with Level 1 predictors			
**Variable**	***F[1,54]***	***P*[a]**	***FDR P*[b]**
FMIN segment 1 - anterior left FA	4.6	**0.037**	0.164
FMIN segment 2 - middle FA	4.0	*0.051*	0.164
FMIN segment 3 - anterior right FA	1.0	0.321	0.482
Left CB - Posterior cluster FA	3.4	*0.073*	0.164
Left UNC FA	0.0	0.900	0.900
Right UNC FA	0.5	0.494	0.556
HC vs Non-depressed BD			
Node clusters identified in Level 1 analysis			
**Variable**	***F[1,68]***	***P*[a]**	***FDR P*[b]**
FMIN middle left cluster FA	0.3	0.576	0.648
Left CB – Anterior cluster FA	0.7	0.422	0.648
Right CB – Anterior cluster FA	0.9	0.345	0.648
Tracts or segments of tracts not associated with Level 1 predictors			
**Variable**	***F[1,68]***	***P*[a]**	***FDR P*[b]**
FMIN segment 1 - anterior left FA	0.4	0.551	0.648
FMIN segment 2 - middle FA	0.6	0.449	0.648
FMIN segment 3 - anterior right FA	0.4	0.508	0.648
Left CB - Posterior cluster FA	3.2	*0.076*	0.648
Left UNC FA	0.8	0.384	0.648
Right UNC FA	0.1	0.769	0.769
Depressed BD vs Non-depressed BD			
Node clusters identified in Level 1 analysis			
**Variable**	***F[1,63]***	***P*[a]**	***FDR P*[b]**
FMIN middle left cluster FA	0.3	0.588	0.799
Left CB – Anterior cluster FA	5.0	**0.030**	*0.270*
Right CB – Anterior cluster FA	2.0	0.169	0.507
Tracts or segments of tracts not associated with Level 1 predictors			
**Variable**	***F[1,63]***	***P*[a]**	***FDR P*[b]**
FMIN segment 1 - anterior left FA	2.8	0.100	0.450
FMIN segment 2 - middle FA	1.0	0.333	0.749

FMIN segment 3 - anterior right FA	0.1	0.710	0.799
Left CB - Posterior cluster FA	0.1	0.824	0.824
Left UNC FA	0.3	0.613	0.799
Right UNC FA	0.2	0.689	0.799

Abbreviations: HC, Healthy Controls; BD, Bipolar Disorder; FDR, False Discovery Rate; FMIN, Forceps Minor; FA, Fractional Anisotropy, CB, Cingulum Bundle; UNC, Uncinate.

[a] P values $\leqslant$ 0.05 are reported in bold characters.

[b] FDR corrected P values.

Table S10. Effects of dMRI movements.

Mean FA	Averaged translation			Averaged rotation		
	r	***P-value***[a]	***FDR P***[a,b]	***r***	***P-value***[a]	***FDR P***[a,b]
Node clusters identified in Level 1 analysis						
FMIN middle left cluster	-0.1	0.508	0.762	<0.1	0.844	0.910
FMIN middle right cluster	<0.1	0.693	0.762	0.1	0.298	0.656
Left CB - Anterior cluster	-0.2	0.150	0.550	-0.2	0.088	0.601
Right CB - Anterior cluster	-0.1	0.669	0.762	-0.2	0.164	0.601
Tracts or segments of tracts not associated with Level 1 predictors						
FMIN cluster 1 - anterior left	-0.1	0.621	0.762	<0.1	0.910	0.910
FMIN cluster 2 - middle	-0.1	0.251	0.690	-0.1	0.551	0.844
FMIN cluster 3 - anterior right	-0.1	0.681	0.762	0.1	0.241	0.656
Left CB - Posterior cluster	-0.2	0.056	0.308	0.1	0.606	0.844
Right CB - Posterior cluster	-0.2	*0.054*	0.308	-0.2	0.110	0.601
Left UNC	<0.1	0.766	0.766	<0.1	0.804	0.910
Right UNC	<0.1	0.693	0.762	-0.1	0.614	0.844

Abbreviations: FMIN, Forceps Minor; CB, Cingulum bundle; UNC, Uncinate; FDR, False Discovery Rate.

[a] P values ≤ 0.05 are reported in bold characters.

[b] FDR corrected P values.

Table S11. Effect of predictors identified in Level 1 analysis in other white matter tracts.

**White matter tracts**	**F**	***P-value***[a]	***FDR P***[a,b]
Forceps Major	3.9	**0.007**	*0.077*
Left anterior thalamic radiation	3.2	**0.019**	0.105
Right anterior thalamic radiation	2.6	**0.046**	0.169
Left corticospinal tract	1.6	0.178	0.392
Right corticospinal tract	0.8	0.551	0.675
Left superior longitudinal fasciculus - temporal	0.8	0.552	0.675
Right superior longitudinal fasciculus - temporal	1.0	0.406	0.638
Left superior longitudinal fasciculus - parietal	2.1	*0.089*	0.245
Right superior longitudinal fasciculus - parietal	0.6	0.651	0.716
Left inferior longitudinal fasciculus	0.3	0.311	0.570
Right inferior longitudinal fasciculus	0.3	0.855	0.855

[a] P values ≤ 0.05 are reported in bold characters.

[b] FDR corrected P values.

Table S12. Correlations between percentage of time taking medications pre-scan and node clusters identified in Level 1 analysis.

Table S12A. Antidepressants and Antipsychotics

Node clusters	Antidepressants			Antipsychotics		
	r	***P-value***[a]	***FDR P***[a,b]	***r***	***P-value***[a]	***FDR P***[a,b]
Node clusters identified in Level 1 analysis						
FMIN middle left cluster	<0.1	0.701	0.936	-0.1	0.563	0.719
FMIN middle right cluster	<0.1	0.916	0.936	0.1	0.518	0.719
Left CB - Anterior cluster	-0.1	0.649	0.936	0.1	0.627	0.719
Right CB - Anterior cluster	-0.2	0.196	0.936	0.1	0.613	0.719
Tracts or segments of tracts not associated with Level 1 predictors						
FMIN segment 1 - anterior left	<0.1	0.891	0.936	<0.1	0.813	0.813
FMIN segment 2 - middle	-0.1	0.350	0.936	0.1	0.369	0.719
FMIN segment 3 - anterior right	0.1	0.565	0.936	-0.1	0.654	0.719
Left CB - Posterior cluster	-0.1	0.492	0.936	-0.1	0.568	0.719
Right CB - Posterior cluster	<0.1	0.805	0.936	0.1	0.419	0.719
Left UNC	<0.1	0.933	0.936	-0.1	0.441	0.719
Right UNC	<0.1	0.936	0.936	-0.2	**0.041**	0.451

Table S12B. Lithium and non-lithium mood stabilizers.

Node clusters	Lithium			Non-lithium mood stabilizers		
	r	***P-value***[a]	***FDR P***[a,b]	***r***	***P-value***[a]	***FDR P***[a,b]
Node clusters identified in Level 1 analysis						
FMIN middle left cluster	-0.3	**0.024**	0.264	-0.2	0.189	0.520
FMIN middle right cluster	<0.1	0.851	0.857	-0.1	0.598	0.805
Left CB - Anterior cluster	-0.2	0.168	0.462	-0.1	0.427	0.805
Right CB - Anterior cluster	-0.2	0.078	0.326	-0.2	0.092	0.337
Tracts or segments of tracts not associated with Level 1 predictors						
FMIN segment 1 - anterior left	-0.2	0.089	0.326	<0.1	0.850	0.850
FMIN segment 2 - middle	<0.1	0.735	0.857	-0.1	0.596	0.805
FMIN segment 3 - anterior right	<0.1	0.857	0.857	-0.1	0.672	0.805
Left CB - Posterior cluster	<0.1	0.688	0.857	-0.1	0.564	0.805
Right CB - Posterior cluster	<0.1	0.855	0.857	<0.1	0.732	0.805
Left UNC	-0.1	0.596	0.857	-0.2	0.091	0.337
Right UNC	-0.1	0.545	0.857	-0.3	**0.020**	0.220

Abbreviations: FMIN, Forceps Minor; CB, Cingulum bundle; UNC, Uncinate; FDR, False Discovery Rate.

[a] P values ≤ 0.05 are reported in bold characters.

[b] FDR corrected P values.

SUPPLEMENTAL FIGURES.

Figure S1. ROC curves.

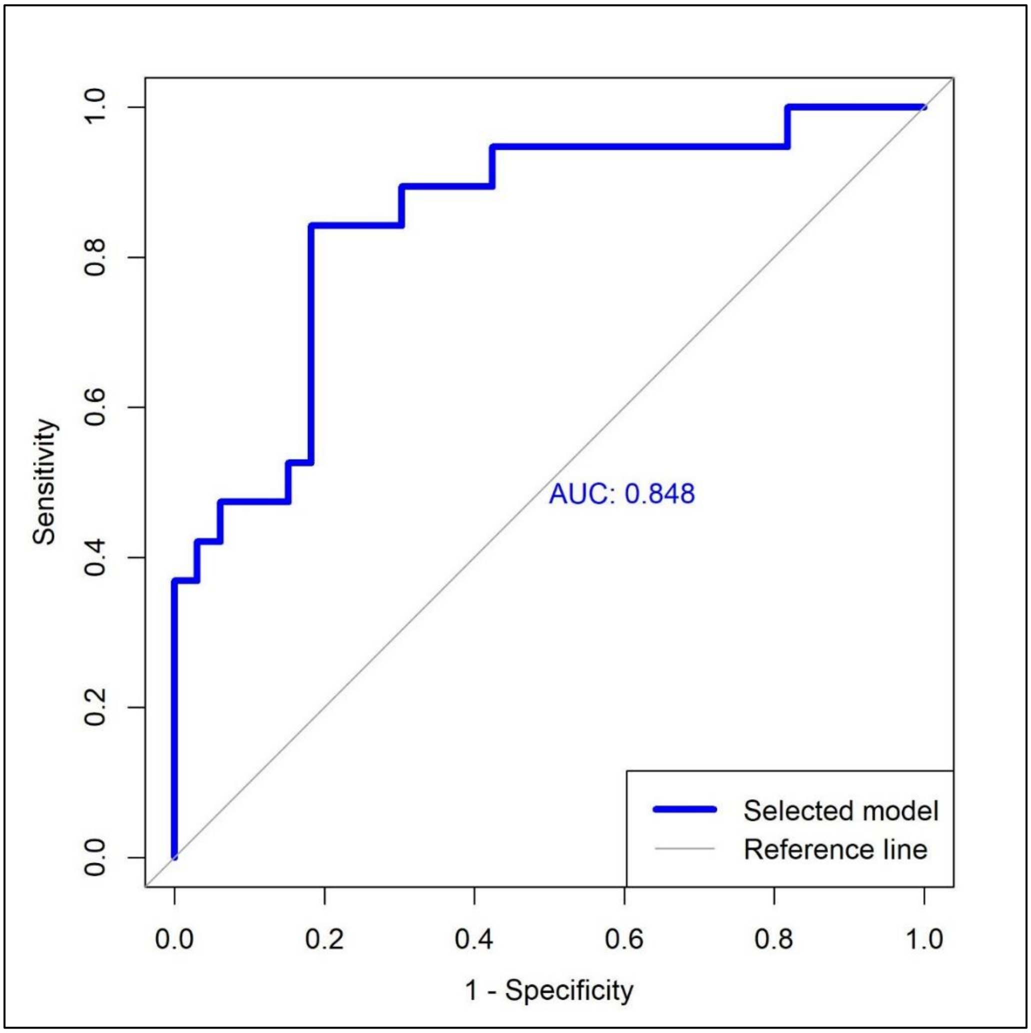

Figure S1 showed the curve for the model identified in Level 2 analysis. This model combines 4 predictors: BIS total score, FMIN middle right cluster (one of the regions associated with pre-scan variables), percentage of time experiencing syndromic depression, and right CB posterior cluster mean FA (segment of the right CB not associated with pre-scan variables). This curve was created by plotting data pairs of sensitivity/specificity and the AUC represents the discriminative ability of the test. Abbreviations: ROC, Receiver Operating Characteristic; AUC, Area Under the Curve; FMIN, Forceps Minor; CB, Cingulum Bundle; FA, Fractional Anisotropy.

SUPPLEMENTARY REFERENCES

1. Andersson, J.L.; Skare, S.; Ashburner, J. How to correct susceptibility distortions in spin-echo echo-planar images: Application to diffusion tensor imaging. *Neuroimage* **2003**, *20*, 870–888.
2. Smith, S.M.; Jenkinson, M.; Woolrich, M.W.; Beckmann, C.F.; Behrens, T.E.; Johansen-Berg, H.; Bannister, P.R.; De Luca, M.; Drobnjak, I.; Flitney, D.E. Advances in functional and structural MR image analysis and implementation as FSL. *Neuroimage* **2004**, *23*, S208–S219.
3. Yendiki, A.; Koldewyn, K.; Kakunoori, S.; Kanwisher, N.; Fischl, B. Spurious group differences due to head motion in a diffusion MRI study. *Neuroimage* **2014**, *88*, 79–90.
4. Yendiki, A.; Panneck, P.; Srinivasan, P.; Stevens, A.; Zöllei, L.; Augustinack, J.; Wang, R.; Salat, D.; Ehrlich, S.; Behrens, T. Automated probabilistic reconstruction of white-matter pathways in health and disease using an atlas of the underlying anatomy. *Front. Neuroinform.* **2011**, *5*, 23.
5. Fischl, B. FreeSurfer. *Neuroimage* **2012**, *62*, 774–781.
6. Behrens, T.E.; Berg, H.J.; Jbabdi, S.; Rushworth, M.F.; Woolrich, M.W. Probabilistic diffusion tractography with multiple fibre orientations: What can we gain? *Neuroimage* **2007**, *34*, 144–155.
